# Big Data use in medical research

**DOI:** 10.1590/S1679-45082018ED4087

**Published:** 2018-09-10

**Authors:** Thiago Gonçalves dos Santos Martins, Ana Luiza Fontes de Azevedo Costa, Thomaz Gonçalves dos Santos Martins

**Affiliations:** 1 Universidade Federal de São Paulo, São Paulo, SP, Brazil; 2 Hospital das Clínicas, Faculdade de Medicina, Universidade de São Paulo, São Paulo, SP, Brazil; 3 Universidade Estácio de Sá, Rio de Janeiro, RJ, Brazil

The current velocity and volume of data generated by websites, electronic sensors and mobile telephones are calculated in exabytes (equal to 1 billion gigabytes) every two days. This amount of data corresponds to what was produced from the begining of the time until 2003. This remarkable figure tends to double every 40 months.^(^ ^1^ ^)^

Big data is a huge set of data that exceeds management by human and requires assistance of computerized and/or analytical processing. Although the volume and velocity in which data are processed in almost real time, quality of data needs working to ensure generation of useful information.

Physicians who study machine learning, attemp to design algorithms that respond and automatically adapt to data, with no need of continuous human intervention. Their goal is to develop artificial intelligence that helps making decisions, given that this large volume of data.^(^ ^2^ ^,^ ^3^ ^)^ These professionals can assist in programming of these machines to assure reliable decision standards. This process leads to reflection upon medical training, and the seeking for new skills and forms of working, which allows adequate selection of information, and enables decision-making in the clinical practice.^(^ ^4^ ^,^ ^5^ ^)^

Multicenter international studies have been conducted in a simpler manner, and currently they involve more participants, and lower costs. Considering this scenario, a huge database with data storaged in different eletronic systems will be required, interconnected with a network and making easier the access to eletronic health records. Data gathering may not be performed without consent, and this fact obliges us to emphasize the need for an ethical debate on adjusting the legislation to this new reality.^(^ ^6^ ^,^ ^7^ ^)^

One of the future challenges will be the process for authorization to the use of data from the internet that are automatically collected daily. New encryption techniques are required to protect patient's private information and ensure data confidentiality. In this type of research, with thousands of hypothesis being simultaneously tested the possibility of a random association should be considered as a significant risk factor.

Physicians have always spent time learning about and how to deal with biased samples.^(^ ^8^ ^)^ The efficient management of an enormous volume of data (Big Data) generated in Medicine can revolutionize the decision-making power of physicians and increase their knowledge about many diseases. However, knowledge on gathering, selecting and analyzing data obtained from real time reporting of results will require a new learning process in Medicine. The purpose is to avoid the analysis of this large quantity of data - with no deep understanding of their context - and to produce only “big noise” (noise is what hinders communication between the emitter and the receptor, in the theory of communication). Therefore, this new learning process demonstrates the importance of cross validation of the data searched, the confirmation of reproducibility of other sets of data and evaluation of possible generalization.^(^ ^9^ ^)^

Big Data is an opportunity to build a large Brazilian database, which may be useful to continuously develop, assess and improve clinical practice guidelines, and to serve as data source for several national and international multicenter studies. In addition, big data represents a tremendous gain in time, money, lives and knowledge.

The future of Medicine is associated to the development of sensors to monitor vital functions and design of new molecules to mark diseases, both combined to supercomputers that are able to process a huge volume of data, and generate a global system to support medical diagnosis.^(^ ^10^ ^)^
